# Assessing differences in the bioaccessibility of phenolics present in two wine by-products using an *in-vitro* model of fish digestion

**DOI:** 10.3389/fvets.2023.1151045

**Published:** 2023-05-02

**Authors:** Francisca P. Martínez-Antequera, Rosalía López-Ruiz, Juan Antonio Martos-Sitcha, Juan Miguel Mancera, Francisco Javier Moyano

**Affiliations:** ^1^Departamento de Biología y Geología, Facultad de Ciencias Experimentales, Universidad de Almería, Almería, Spain; ^2^Departamento de Biología, Facultad de Ciencias del Mar y Ambientales, Instituto Universitario de Investigación Marina (INMAR), Campus de Excelencia Internacional del Mar (CEI·MAR), Cádiz, Spain; ^3^Research Group “Analytical Chemistry of Contaminants”, Department of Chemistry and Physics, Research Centre for Mediterranean Intensive Agrosystems and Agri-Food Biotechnology (CIAIMBITAL), Agrifood Campus of International Excellence, ceiA3, University of Almeria, Almeria, Spain

**Keywords:** polyphenols, UHPLC-Q-Orbitrap-MS, wine by-products, fish species, *in vitro* digestion

## Abstract

Increasing attention is currently being paid to the protective role of polyphenols in health and oxidative status in fish. For this reason, the potential use of different natural sources of such compounds, like wine by products, is under study. One key step required to gain a better understanding on the biological roles of polyphenols for a given species is to assess the different factors affecting their digestive bioaccessibility, and a great number of such studies is based in the use of *in vitro* digestion models. In the present study the potential digestive bioavailability of the phenolic compounds present in wine bagasse and lees was evaluated for two fish species showing great differences in their digestive phisyiology: the omnivorous gilthead sea bream (*Sparus aurata*) and the herbivorous flathead grey mullet (*Mugil cephalus*). The study was developed using *in vitro* models adapted to simulate their digestion and a factorial experimental design that simultaneously evaluated the effects of the ingredient used as source of polyphenols, presence or absence of feed matrix, fish species and digestion time. The release of the phenolic compounds was evaluated using ultra-high performance liquid chromatography (UHPLC) coupled to high resolution mass spectrometry (HRMS) detection. Both the presence of feed matrix and the type of wine by-product showed a significant effect on the digestive release of both total and specific types of polyphenols while fish species showed to be significant only for some specific compounds, like eriodyctiol or syringic acid. The time of digestion was not identified as a statistically significant factor in the release of phenolic compounds due to the great variability in the patterns observed that were classified as early, sustained and late. The observed great variations in the patterns of release of different types of phenolic compounds with time suggest an important effect of gut transit rates on the net bioavailability of a given phenolic compound in the live fish. The present study is, to our knowledge, the first one on which an *in vitro* approach was applied to assess to what extent the possible complexation of wine polyphenols present in wine by-products with either digestive enzymes or components of the feed matrix could limit their bioaccessibility if included in diets of two different fish species.

## 1. Introduction

Increasing attention is being paid to the inclusion of biologically active ingredients in aquaculture feeds that can benefit fish health and resistance to different stressors (changes in environmental parameters -temperature, salinity, etc., alterations in water quality, manipulation, etc.) as well as disease outbreaks. Within this context, it is of great interest to investigate the potential use of bioactive molecules with antioxidant and immunostimulatory functions present in a great number of agro-industrial by-products. Indeed, the valorization of some of these materials as a source of active compounds would be in line with the principles of the circular economy. In this sense, by-products from the winemaking process represent a cheap and exceptionally rich source of valuable compounds that could be used as natural additives and functional ingredients (1–5). Wine by-products have traditionally been used in feeding terrestrial animals mainly as a source of fiber, carbohydrates and minerals, but more recently, different studies have highlighted their role as a source of different chemical compounds, mostly phenolics, with positive effects in the production of pigs, poultry or ruminants (6–8). In the case of fish, the few studies published to date suggest that grape polyphenols may also produce beneficial effects. The more remarkable are the prevention of liver diseases related to oxidative stress (9), reduction in the deterioration of cellular energy homeostasis (10), improvement in the growth and feed digestibility (11) and changes in the composition of the intestinal microbiota (12).

The study of the intestinal absorption and bioavailability of dietary phenolic compounds in humans and animals is a complex issue that involves several factors. In the one hand, they are influenced by physicochemical properties of the different molecules (chemical structure, molecular size, configuration, lipophilicity, solubility, pKa) and in the other by their interactions with other components of the digesta (food/feed matrix and digestive enzymes), as well as with the colonic microbiota. All those factors determine great differences in the rates of absorption and hence in bioavailability of phenolics present in different vegetable sources (plants and fruits, either raw or processed and also in by-products) and hence in their observed biological effects. In the case of humans, and to a lesser extent in terrestrial animals, many of the above mentioned aspects have been extensively addressed using a wide array of *in vitro* assays (13–15). Although there is a great difference between biological properties of polyphenols observed *in vitro* and their bioactivity *in vivo*, simulations of the physiological conditions present in the digestive tract become a valuable tool that helps to reach a greater understanding on how such factors may influence potential bioavailability of phenolics (16). *In vitro* digestive simulations of aquatic animals have been used with different purposes; the evaluation of protein quality of feed ingredients (17, 18), the study of factors affecting the efficiency of enzyme hydrolysis in the digestion (19) or the effect of the digestive biochemistry on toxic compounds (20). Nevertheless, to date they have not been used to evaluate the potential beneficial effects of including phenolic compounds in diets.

A key aspect required for a proper evaluation of the release of phenolic compounds in experiments simulating digestion is to apply methodologies allowing and accurate quantitative and qualitative detection of the highly diverse profiles that can be obtained.

The analysis of phenolic compounds present in wine and its by-products has been routinely developed using liquid chromatography (LC) coupled to diode array detection (DAD), due to its high sensitivity and easy operation (21, 22). Nevertheless, some problems occur when ultraviolet (UV) spectrum is studied, since the UV spectrum of phenolic compounds is quite similar, being the identification ambiguous. Due to this, the best option for the analysis of phenolic compounds is the use of LC coupled to mass spectrometry (MS) using tandem MS/MS stages and an Electrospray Ionization (ESI). Of course, due to the physical–chemical properties of phenolics the ionization mode was in positive and negative (22–25). In addition, in the last years the use of high-resolution mass spectrometry (HRMS) has emerged as a revolutionary way for screening samples in a short time to obtain a complete characterization of them. In this case, a complete profile of phenolic compounds can be achieved using the capacity of full scan acquisition, exact mass resolution and the used of HRMS spectral libraries that provide the possibility to detect 1000s of compounds without using analytical standards (23).

Considering all the aforementioned, the objective of the present study was to assess how different factors can affect the potential bioavailability of the phenolic compounds present in two types of wine by-products (bagasse -WB- and lees -WL-) when provided in the feed of two fish species. The species chosen were the gilthead sea bream (*Sparus aurata*) and the flathead grey mullet (*Mugil cephalus*) and they were selected considering that both of them are commonly aquacultured species that could potentially benefit from the protective effect of a dietary reinforcement in phenolics. Also, this may help to illustrate the expected differences linked to particular features of their digestive physiology (mainly determined both by the amount and types of digestive enzymes and by the presence of an acid stage in the digestion of seabream that is absent in the case of mullet).

The study was based in (a) the use of an *in vitro* model adapted to simulate the digestion of both fish species; (b) a factorial experimental design, oriented to evaluate the effect of different factors involved in the digestion and (c) an accurate analytical methodology of the phenolic compounds based in using ultra-high performance liquid chromatography (UHPLC) coupled to high resolution mass spectrometry (HRMS) detection. The study was run as a preliminary step to the development of an *in vivo* test currently in progress, since we expect that results obtained would provide valuable information related to the selection of the most suitable ingredient, as well as on the biological responses derived from its dietary inclusion.

## 2. Materials and methods

### 2.1. Origin and characterization of the wine by-products (bagasse and lees)

Two types of by-products were obtained from red grape varieties: (i) wine bagasse (WB) came from an artisanal winery located in Fondón (Almería. Spain) and (ii) wine lees (WL) came from a winery located at Chiclana de la Frontera (Cádiz. Spain). Upon collection the two products were stored at −20°C until processed or used for the different analysis. Processing consisted in the case of WB in a drying at low temperature (28°C) followed by milling until obtaining a fine powder. The WL were simply thawed prior to being used in liquid form. The composition of both WB and WL is detailed in Table 1.

**Table 1:** Proximal composition and total phenolics in red wine bagasse (WB) and lees (WL).

Proximal composition (g/kg)	WB			WL		
Total fat	54	±	8	27	±	4
Saturated fatty acids	8	±	2	8	±	1
Total carbohydrates	320	±	60	130	±	20
Reducing sugars	162	±	7	3.4	±	2.8
Dietary fiber	470	±	90	480	±	100
Crude protein	83	±	12	180	±	20
Total minerals	35	±	7	160	±	30
Na		<	0.05	0.11	±	0.02
Bioactive compounds						
Total phenolics	53.8	±	0.7	21.4	±	0.9
Antioxidant capacity (μmol TEAC/g d.m.)	337.4	±	14.1	134.4	±	16.0

### 2.2. Wine by-products experiments

#### 2.2.1. Experiment 1

The assay was aimed to assess the potential inhibitory effect of phenolics present in WB on the digestive proteases of one of the species used in the study; the gilthead sea bream (*Sparus aurata*). It was developed following a protocol previously applied by our group (26). In brief, the assays were carried out incubating for 1 h a fixed amount of enzyme extracts obtained from the stomach or intestine of fish specimens in the presence of variable amounts of WB. After this time, residual protease activity was measured and expressed in relation to a control on which the enzymes were incubated in the presence of distilled water. The enzyme extracts were prepared by manual homogenization of tissues obtained after dissection of fish weighing about 100 g, followed by cold centrifugation (20,000 x g; 4°C) and separation of clear supernatants. After this process, the activities of stomach and intestinal proteases were determined in the extracts; acid protease was measured at pH 2.5 using hemoglobin as substrate (27) while alkaline protease was measured at pH 8.5 using casein (28).

#### 2.2.2. Experiment 2

The objective was to evaluate changes in the potential digestive bioavailability of phenolics present in both WB and WL either when used pure or included in a feed matrix. Such experimental feed matrix was prepared using some pure ingredients in proportions (in g/100 g) reflecting the composition of a standard fish feed: bovine albumin (45%), sunflower oil (18%), potato starch (10%) and carboxy methyl cellulose (15%). The matrix was prepared by mixing the ingredients with some distilled water in order to obtain a moist paste that was used as substrate to which WB or WL were added to reach a 10% in dry weight. *In vitro* simulation of fish digestion was carried using a protocol developed by our group, based on the use of semi-permeable membrane bioreactors (29). Each device consists of two chambers separated by a membrane of 3,500 kDa MWCO (ZelluTrans/Roth). Fish enzyme extracts and substrates are placed in the upper chamber and maintained under continuous agitation using a magnetic stirrer. To develop the acid phase of digestion (only in the case of *S. aurata*) the upper chamber contained the desired substrate dissolved in water and adjusted to pH 4.0 by addition of a few drops of HCl 0.1 M, as well as the crude enzyme extract from the stomach of the selected species while the lower chamber contained distilled water. During the alkaline phase, pH of the upper chamber was raised to pH 8.2 using borate buffer (0.1 M supplemented with 20 mM CaCl_2_, sodium taurocholate 45 μM and NaCl 50 mM) prior to the addition of the intestinal enzyme extracts. Products released during the reaction time and passing across the membrane into the lower chamber can be recovered at different time intervals by a constant flow of the same alkaline buffer and used to determine the release of products contained in the substrate (phenolics in our case). The complete arrangement (formed by several experimental units) is maintained within a thermal chamber set at the desired temperature.

Digestive enzyme extracts of seabream were obtained after dissection from two different sections (stomach and proximal intestine) of 15 individuals of approximately 50 g, while in the case of mullets they were obtained only from the intestinal portion of 5 adult specimens of approximately 3 kg. Specimens of both species were supplied by Central Research Services in Marine Cultures (SCI-CM, Operational Code REGA ES11028000312) of the University of Cádiz. The extracts were prepared by mechanical homogenization of the tissues in distilled water (1:10 w/v) followed by centrifugation (3,220 × g, 20 min, 4°C). The supernatant was then filtered through a dialysis system with a MWCO of 10 kDa (Pellicon XL, Millipore) and the concentrated extracts were freeze-dried until being required for the assays. Activities of acid and alkaline proteases were determined as indicated in Experiment 1. The values of protease activities were used as indicators to estimate the number of extracts required to provide physiological enzyme:substrate ratios in the assays developed for each species. These were calculated considering, on one hand, the average total production of enzyme measured in a few fish in relation to their live weight and on the other, the average intake per meal of fish of such size, obtained from commercial ration tables. This information resulted in values of 33.3 and 16.7 U/mg protein for stomach and intestinal digestion in seabream and 18.3 U/mg protein for intestinal digestion in mullet.

Different experiments were developed following a factorial design that simultaneously evaluated the effect of type of product, presence or not of feed matrix, fish species and digestion time. Details of this design are presented in Table 2 and the combination of the different factors resulted in a set of 16 different runs.

**Table 2:** Conditions used and parameters measured on the *in vitro* experiment aimed to test the effect of feed matrix in the bioavailability of wine polyphenols.

Fish species	Gilthead seabream (*Sparus aurata*)
	Flathead mullet (*Mugil cephalus*)
Factors evaluated	Feed matrix (presence/absence)
	Type of product (bagasse/lees)
	Fish species (seabream/mullet)
	Time of intestinal digestion (3/6 h)
Parameters measured in the digestate	Type and amount of specific phenolic compounds (UHPLC-Q-Orbitrap-MS)

### 2.3. UHPLC-Q-Orbitrap-MS analysis

Once the hydrolysis experiments were conducted, the obtained samples were analyzed to determine the profile of the phenolic compounds released. Sample treatment and UHPLC-Q-Orbitrap-MS analysis was based on a previous study developed in the research group by (24). A previous extraction was carried out by mixing 2 mL of the dialysate samples with a mixture composed of methanol:water (80:20, *v/v*). After they were shaken for 1 min in a vortex and 2 h on a rotary shaker. They were then centrifuged at 5,000 rpm for 10 min to collect the supernatant that was diluted in a proportion 1:10 (*v/v*) with extraction solvent. Once the samples were extracted, they were injected and analyzed by UHPLC-Q-Orbitrap-MS. The chromatographic separation was performed on a Vanquish Flex Quaternary LC (Thermo Fisher Scientific, San Jose, CA, United States) was used equipped with a reverse-phase C18 column, Hypersil Gold (100 mm × 2.1 mm, 1.9 μm, Thermo Fisher Scientific) at flow rate of 0.2 mL/min. The compounds were separated with gradient elution using water (A) and methanol (MeOH) (B) containing both 0.1% formic acid as eluents. The step gradient was as follows: 0–1 min 95% of A; then, it was linearly decreased to 70% in 2.5 min, to 0% in 2.5 min and remained constant during 8 min. Finally, it increased to 95% in 2 min and remained constant during 5 min. The total running time was 20 min. The injection volume was 10 μL and column temperature was 30°C (24).

The LC system was coupled to a hybrid mass spectrometer Q-Orbitrap Thermo Fisher Scientific (Q-Exactive™, Thermo Fisher Scientific, Bremen, Germany) using ESI (HESI-II, Thermo Fisher Scientific, San Jose, CA, United States) in positive and negative ion mode. ESI parameters were as follows: spray voltage, 4 kV; sheath gas (N_2_, 95%), 35 (arbitrary units); auxiliary gas (N_2_, 95%), 10 (arbitrary units); S-lens RF level, 50 (arbitrary units); heater temperature, 305°C, and capillary temperature, 300°C. The mass spectra were acquired employing four alternating acquisition functions: (1) full MS, ESI +, without fragmentation (the higher collisional dissociation (HCD) collision cell was switched off), mass resolving power = 70,000 Full Width at Half Maximum (FWHM); AGC target = 1e6, scan time = 250 ms; (2) full MS, ESI -, without fragmentation (the higher collisional dissociation (HCD) collision cell was switched off), mass resolving power = 70,000 FWHM; AGC target = 1e6, scan time = 250 ms; (3) data independent analysis (DIA), ESI +, setting higher energy collisional dissociation (HCD) on, and collision energy = 30 eV, mass resolving power = 35,000 FWHM, scan time = 125 ms; (4) DIA, ESI - (setting HCD on, and collision energy = 30 eV), mass resolving power = 35,000 FWHM, scan time = 125 ms. The mass range in the full scan MS experiments was set to m/z 50–750. LC chromatograms were acquired using the external calibration mode.

The raw files obtained from each analysis were processed using the software Trace Finder 4.0 (Thermo Fisher Scientific, Les Ulis, France) with an in-house database composed of around 100 polyphenols. This database involved the name of the compounds and their molecular formula, theoretical exact mass of the precursor ion and theoretical exact mass of two fragments. Moreover, full-scan data of each sample was carefully studied using Xcalibur TM version 3.0, with Qualbrowser to monitor the spectra of the detected compounds.

### 2.4. Statistics

The values of enzyme inhibition were subjected to arcsin transformation prior to be evaluated by one-way ANOVA followed by a Fisher’s LSD test at a confidence level of 95%. The design and further evaluation of the data of the factorial experiment (relative surface of the different peaks corresponding to each polyphenol, obtained in the UHPLC- -MS assays) were carried out using the DOE module of the Minitab software 17 (Minitab Inc., State College, PA. United States).

## 3. Results

**Figure 1:**
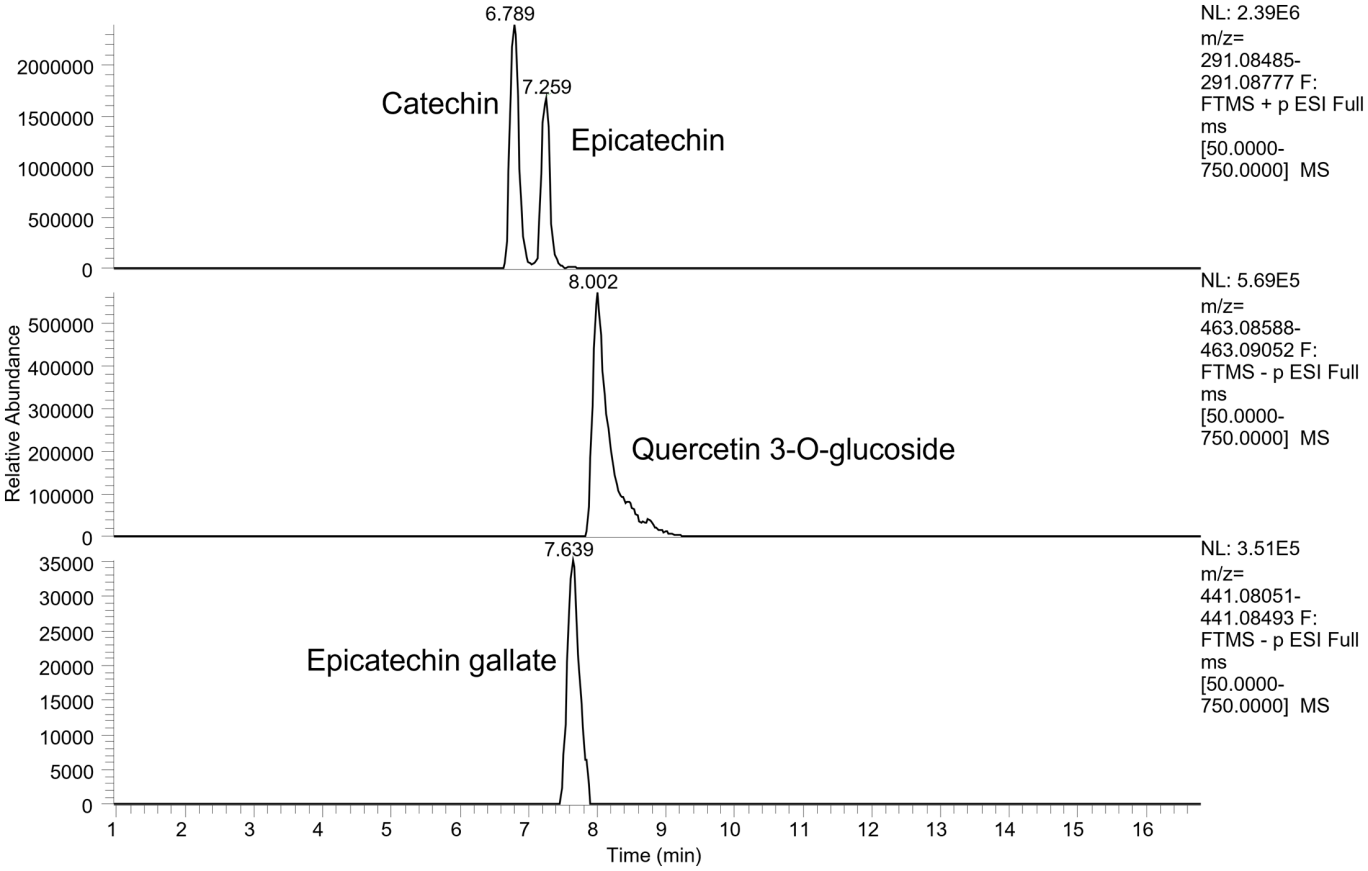
Example of an extracted ion chromatogram of the flavonols and flavanol catechin, epicatechin, quercetin 3-O-glucoside and epicatechin gallate in samples of WB.

A total of 13 main phenolic compounds were identified by UHPLC-Q-Orbitrap-MS for wine by-products in the digested samples, used in the present study, being classified in relation to their chemical structure as detailed in Table 3. In the case of WB it showed a major presence of flavan-3-ols (catechin, gallocatechins and procyanidins), flavonols (kaempferol, quercetin) and hydroxybenzoic acids like syringic and chlorogenic acids while the phenolic profile of WL showed the presence of flavanols such as quercetin, quercitrin and kaempferol, as well as flavanols like catechin, epicatechin, and procyanidin B2. Figure 1 shows an example of extracted ion chromatogram of the flavonols and flavanols catechin, epicatechin, quercetin 3-O-glucoside and epicatechin gallate.

**Table 3:** List of the main phenolic compounds identified in the wine by-products used in the study by UHPLC-Q-Orbitrap-MS.

**Name**	**Type**	**Chemical formula**	**Precursor ion (*m/z*)**	**Mass error (ppm)**	**Product ion (*m/z*)**	**Retention time (min)**	**Ionization mode**
Catechin	Flavanol	C_15_H_14_O_6_	291.08631	−2.64	139.03895	6.99	Positive
Epicatechin (EC)	Flavanol	C_15_H_14_O_6_	291.08631	−2.85	123.04491	7.45	Positive
Epicatechin gallate (ECG)	Flavanol	C_22_H_18_O_10_	441.08272	−4.67	169.01304	2.27	Negative
Epigallocatechin (EGCG)	Flavanol	C_15_H_14_O_7_	305.06668	−0.19	255.92270	2.93	Negative
Kaempferol-3-O-glucoside	Flavonol	C_21_H_20_O_11_	447.09328	−0.89	255.02924	8.42	Negative
Quercetin 3-O-glucoside	Flavonol	C_21_H_20_O_12_	463.08820	0.19	302.03696	8.25	Negative
Quercetin-3-O-rhamnoside (quercitrin)	Flavonol	C_21_H_20_O_11_	447.09328	−0.18	230.98517	8.40	Negative
Quercetin-3-O-rutinoside (rutin)	Flavonol	C_27_H_30_O_16_	609.14611	−0.48	301.03474	8.20	Negative
Eriodyctiol	Flavanone	C_15_H_12_O_6_	287.05611	−3.68	151.00235	3.48	Negative
Naringenin	Flavanone	C_15_H_12_O_5_	271.06120	−0.61	119.04879	8.82	Negative
Chlorogenic acid	Phenolic acid	C_16_H_18_O_9_	353.08781	−0.38	191.05610	7.55	Negative
Syringic acid	Phenolic acid	C_9_H_10_O_5_	197.04555	−2.57	123.00734	7.96	Negative
Procyanidin B1	Non hydrolysable tannin	C_30_H_26_O_12_	577.13515	−0.46	289.07154	6.64	Negative

**Figure 2:**
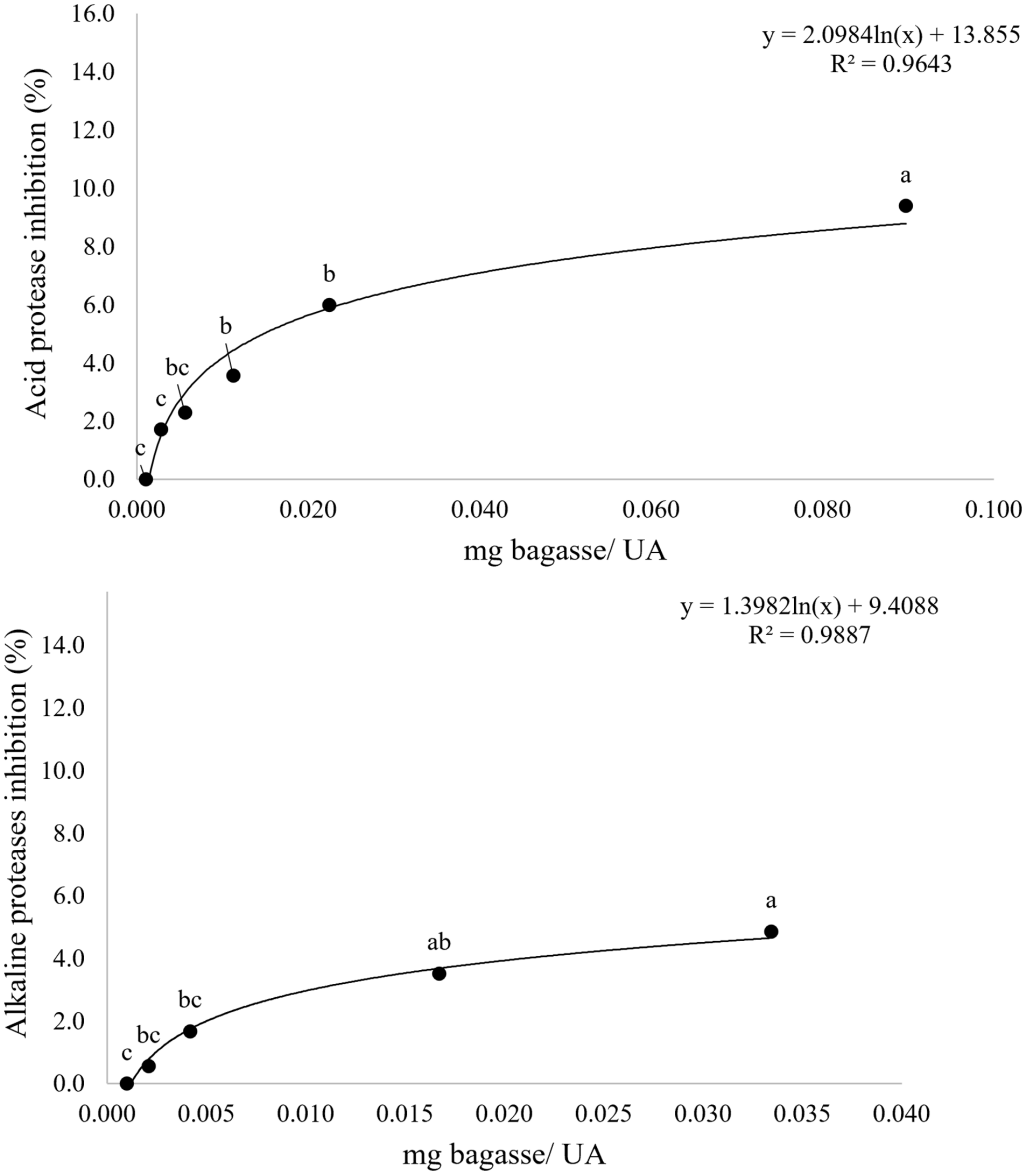
Inhibition curve of acid and alkaline protease activity obtained after 1 h incubation of digestive extracts of gilthead seabream in the presence of increasing concentrations of wine bagasse. Each point is the mean of three replicate measures. Points not sharing a common letter are statistically different with *p* < 0.05.

**Figure 3:**
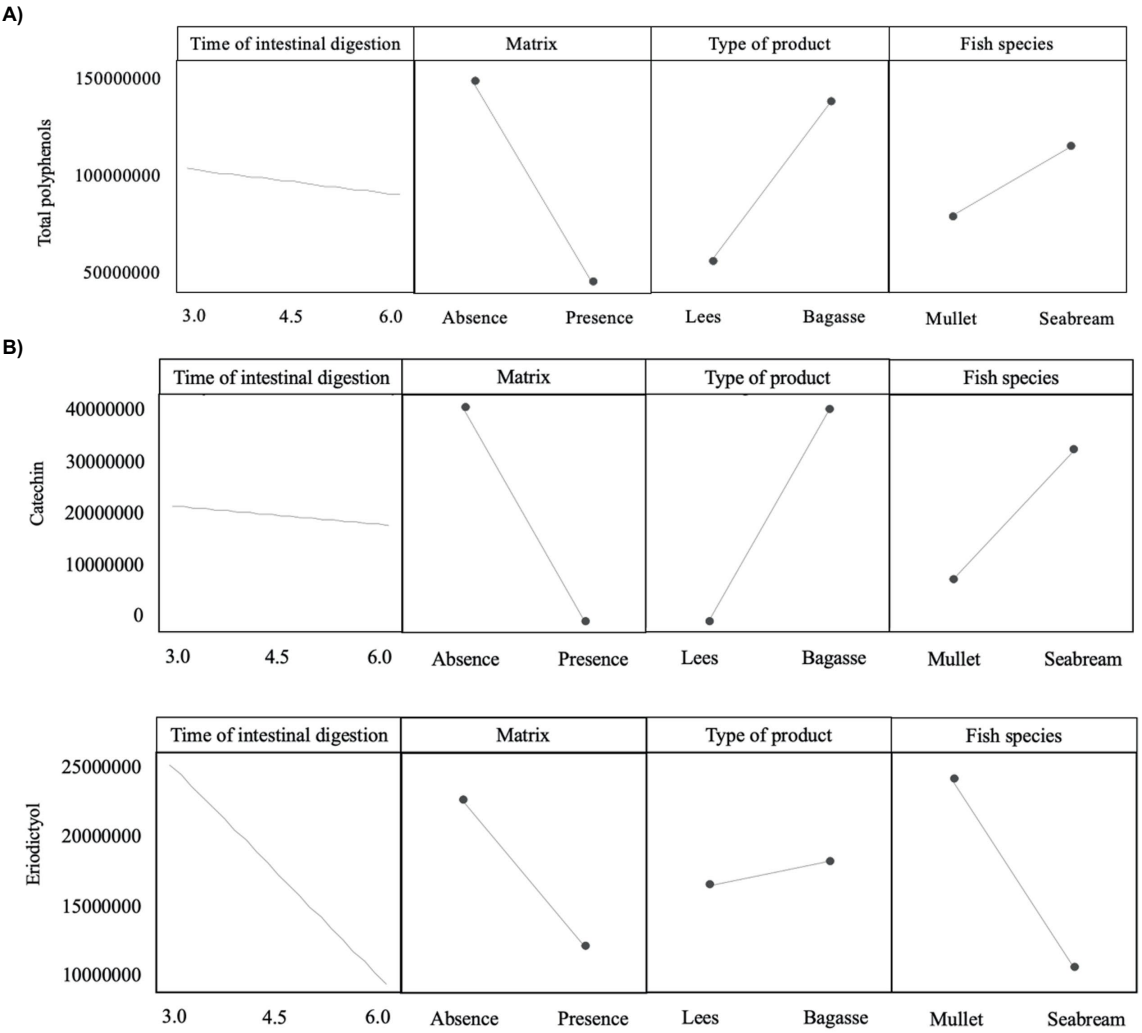
Plot of the main effects evaluated on the release of **(A)** total polyphenols and **(B)** two specific types of phenolic compounds (catechin and eriodyctiol).

**Figure 4:**
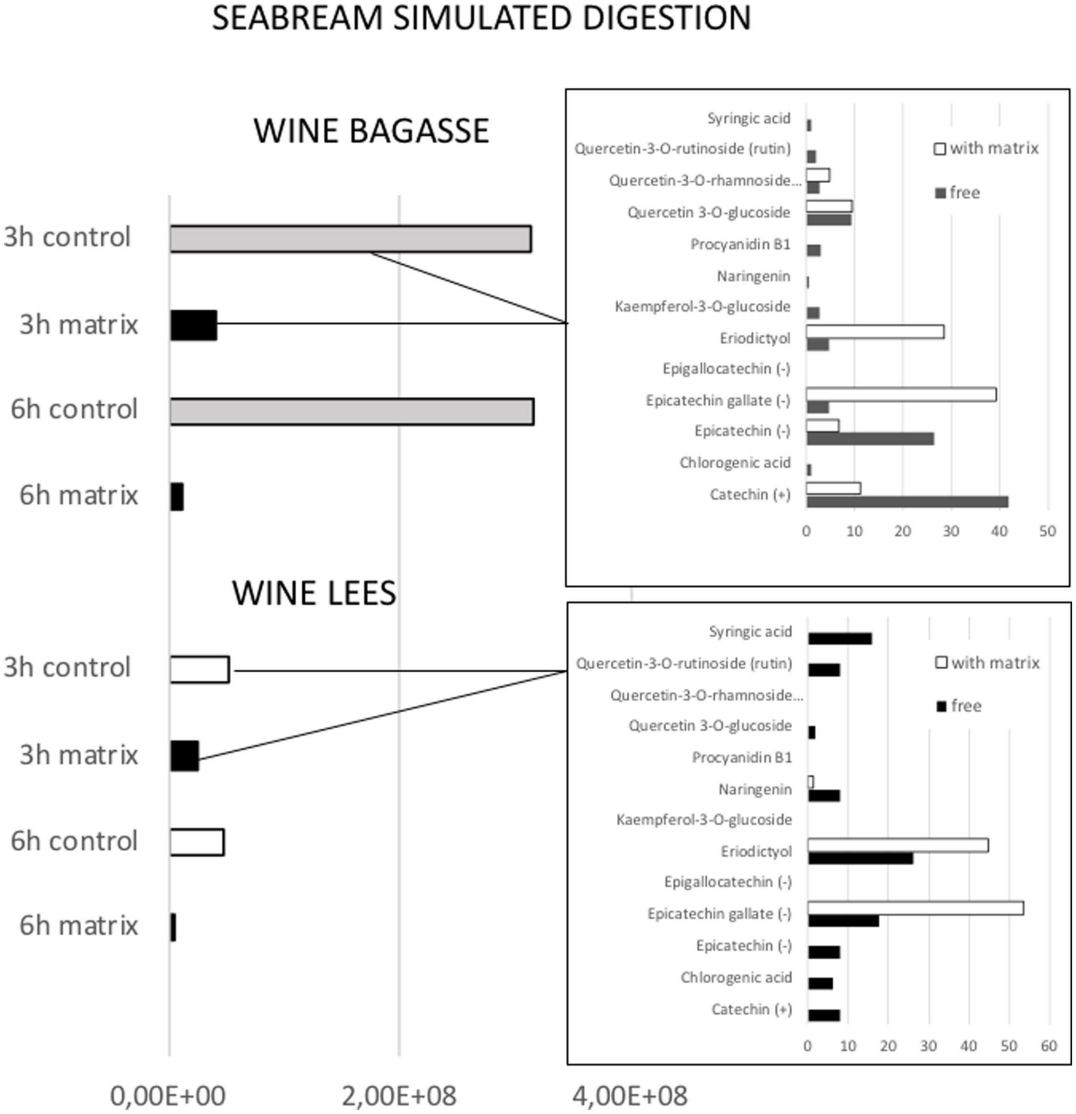
Effect of feed matrix on the release of total phenolic compounds from either wine bagasse or lees, measured at two different digestion times (3 and 6 h) during simulated digestion of seabream. Values are expressed as the sum of all peak areas detected in samples measured by UHPLC. The release of specific types of phenolics after 3 h digestion of either bagasse or lees are shown in the small graphs, being values expressed as % of the total amount of phenolics measured in the presence or absence of feed matrix.

**Figure 5:**
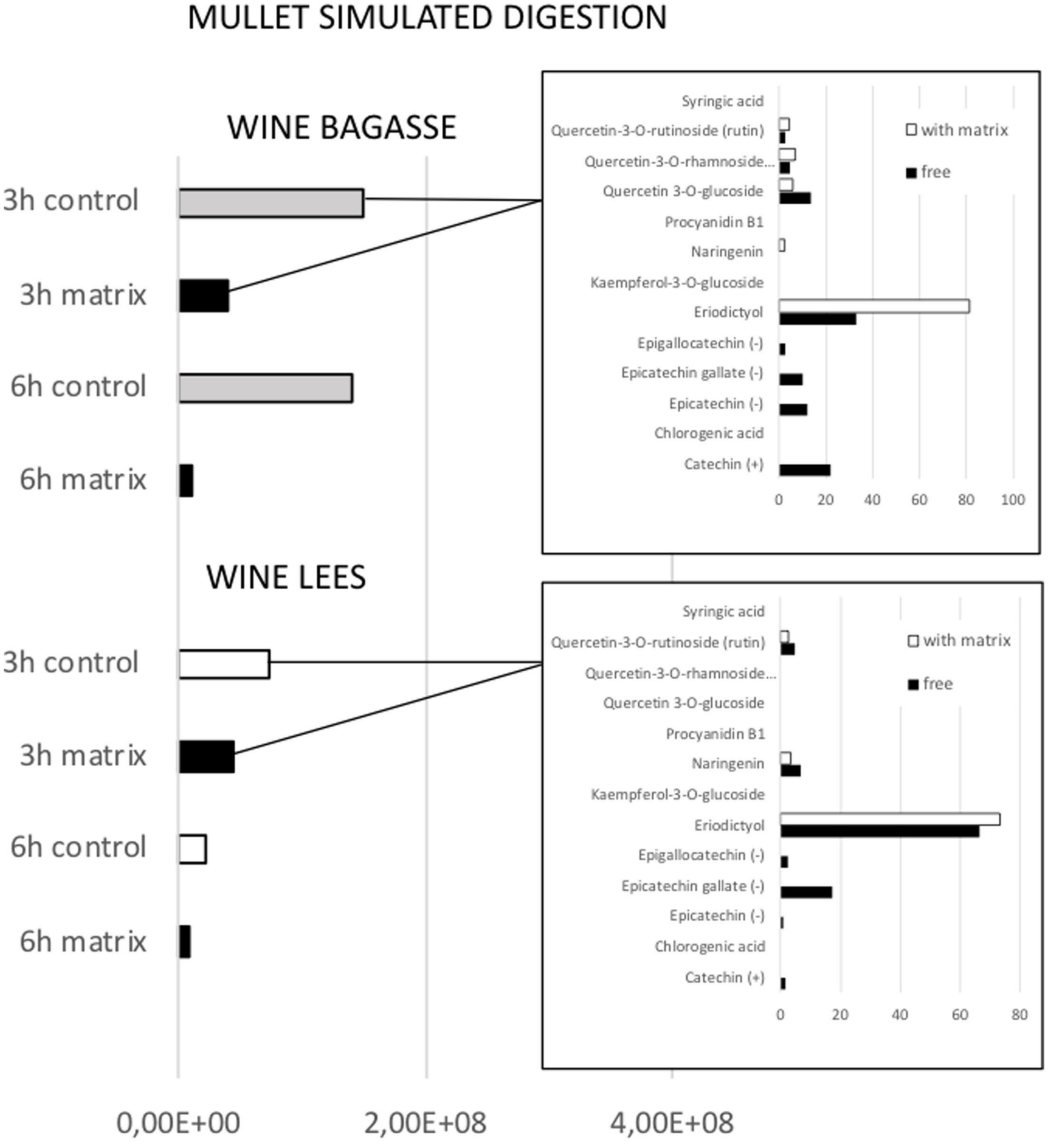
Effect of feed matrix on the release of total phenolic compounds from either wine bagasse or lees, measured at two different digestion times (3 and 6 h) during simulated digestion of flathead mullet. Values are expressed as the sum of all peak areas detected in samples measured by UHPLC. The release of specific types of phenolics after 3 h digestion of either bagasse or lees are shown in the small graphs, being values expressed as % of the total amount of phenolics measured in the presence or absence of feed matrix.

**Figure 6:**
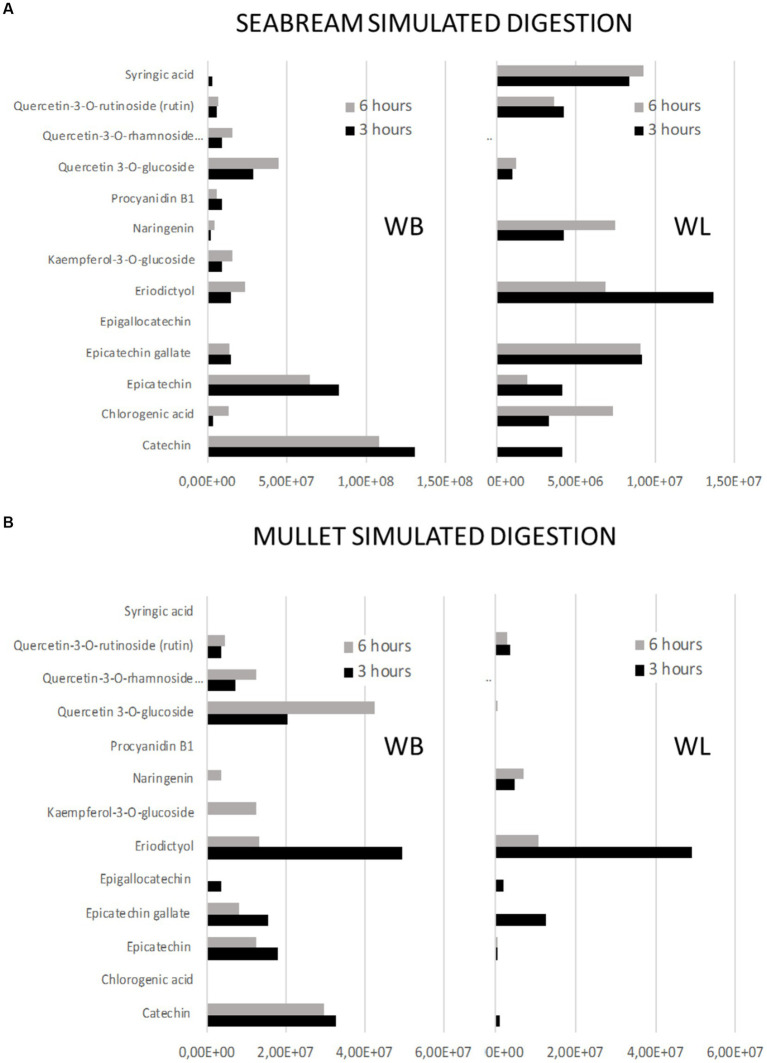
Profiles of release of phenolic compounds from either wine bagasse (WB) or lees (WL) measured at two different time moments (3 and 6 h) during simulated digestion of **(A)** seabream and **(B)** flathead mullet. Values are expressed as the sum of all peak areas detected.

The results of the inhibition of activity of the proteases present in stomach and intestine of sea bream (*S. aurata*) when incubated in the presence of WB are detailed in Figure 2. The values reached in both cases pointed to a maximum reduction of either 8 and 4% of the activities of stomach or intestinal proteases, respectively. Results obtained in assays simulating digestive hydrolysis of matrix including any of the two wine byproducts by fish enzymes are summarized in Table 4 and Figures 3–6. As shown in Table 4, the presence of feed matrix was the only factor with a significant effect on the digestive release of both total and specific types of polyphenols (from *p* < 0.001 to *p* < 0.037 for different compounds). The type of wine by-product was also a significant factor affecting the digestive release of most polyphenols and in consequence, the interaction of both factors showed also to be significant for most of them. In contrast, fish species was not a significant factor affecting the total release of polyphenols, although it showed to be significant for some specific compounds, like eriodyctiol or syringic acid.

**Table 4:** Significance of the evaluated factors on the amounts of total and some selected phenolic compounds released from wine by-products under conditions simulating fish digestion.

	TOTAL	Catechin	Eriodyctiol	Epicatechin	Kaempferol-3-glucoside	Quercentin-3-o-glucoside	Syringic acid
				*p*-value			
Model	0.008	0.031	0.007	0.040	0.053	0.003	0.082
Lineal	0.003	0.017	0.003	0.023	0.032	0.001	0.053
Total digestion time (TDT)	0.438	0.751	0.002	0.664	0.144	0.112	0.790
Presence of matrix (M)	**0.001**	**0.012**	**0.011**	**0.016**	**0.021**	**0.001**	**0.037**
Type of wine by-product (WP)	**0.004**	**0.012**	0.547	**0.019**	**0.021**	**0.001**	0.099
Fish species (FS)	0.076	0.065	**0.004**	0.058	0.344	0.389	**0.037**
Interactions	**0.031**	0.073	**0.024**	0.088	0.108	**0.015**	0.142
TDT * M	0.937	0.670	0.508	0.593	0.144	0.078	0.790
TDT * WP	0.498	0.849	0.192	0.733	0.144	0.125	0.619
TDT * FS	0.389	0.827	**0.002**	0.823	0.607	0.803	0.790
M * WP	**0.005**	**0.014**	0.300	**0.021**	**0.021**	**0.001**	0.099
M * FS	0.053	0.075	0.515	0.066	0.344	0.720	**0.037**
WP * FS	0.056	0.071	0.368	0.075	0.344	0.481	0.099
*R*-square	95.71%	92.28%	96.00%	91.32%	90.16%	97.06%	88.03%
*R*-square fitted	87.12%	76.84%	88.0%	73.95%	70.48%	91.18%	64.09%

Significant (*p* < 0.05) factors and interactions are indicated in bold letters.

The magnitude and trend of the above mentioned significant effects can be clearly evaluated in Figures 3–5. The plot of the main effects presented in Figure 3 indicates that the presence of feed matrix significantly decreased the release of phenolic compounds, irrespective of the species and type of product, and that total amount of available phenolics was significantly higher in WB. Also, the effect of fish species (determined both by the source of enzymes and by the presence of an acid stage in the digestion of seabream, that was absent in the case of mullet) was the opposite for catechin and eriodyctiol, being the release of this latter significantly influenced by the time of digestion but not in the case of catechin. These points, as well as some additional aspects like the different (and sometimes opposite) effect of matrix on the release of specific phenolics present in both WB and WL when digested by the enzymes of the two fish species can be appreciated in more detail in Figures 4, 5. As shown in the mentioned figures, fish species significantly influenced the profiles of phenolics release, being the differences both quantitative and qualitative. Quantitative differences were evident since, in absence of matrix, the simulated digestion of mullet released around 50% less compounds from WB than that of seabream, while in contrast, the total amount released from WL was 40% higher. Nevertheless, such differences disappeared when any of the two by-products were included in the feed matrix. Regarding the effect on specific compounds, it was noticed that seabream enzymes released mainly catechin, epicatechin and quercetin from WB, while in the case of mullet, besides quercetin and catechin, the main product released was eriodyctiol. In a similar way, kaempferol was practically the only product released from WL by the digestion of mullet, while in the case of sea bream a more complex profile including naringenin, epicatechin or syringic acid was observed. Also, in both species, the diversity of phenolic compounds released after inclusion of WL in the feed matrix was significantly reduced when compared to that produced by WB.

The time of digestion was not identified as a statistically significant factor in the release of phenolic compounds because some of them showed opposite patterns of release as a function of time. Nevertheless, a detailed analysis of the peaks identified in the profiles (Figure 6) offers important information, allowing to identify three different patterns of release:

- *Early* (when most part of the compound was measured in the digestate after 3 h of intestinal digestion). This would be the case of eriodictyol released for WB in the digestion of mullet, or from WL in the digestion of sea bream and for kaempferol and epigallocatechin after the digestion of WL by the mullet.
- *Sustained* (when significant amounts of the product are measured both 3 h and 6 h after the digestion). This was the case of catechins and epicatechins present in WB or of syringic acid in WL when digested by both species.
- *Late* (when the compound was detected only or mainly after 6 h of alkaline digestion). This was the case for quercetin present in WB for both fish species or for chlorogenic acid or naringenin present in WL when digested by sea bream.

## 4. Discussion

The profile of phenolic compounds identified in both WB and WL was similar to that described previously by other authors (30–32). Nevertheless, important differences between the WB and WL were evidenced during the *in vitro* study of release. The phenolic profile of WL depends on the type of crushed grapes and other factors that are present during wine production (i.e., maturation time, material of the barrel, etc), since they are transferred to the yeast due to the adsorption capacity of their cell wall (33). WL have been pointed out as a good source of flavanols such as quercetin, quercitrin, kaempferol, and myricetin although also flavanols, namely catechin, epicatechin, and procyanidin B2, were also identified (34).

An accurate estimation of the extent and relevance of potential positive effects associated to the inclusion of wine polyphenols in fish feeds needs a detailed assessment of the bioavailability of the different compounds. This is required to correctly establish dose–response relationships. Bioavailability, defined as the fraction of a nutrient or compound released from the food/feed matrix after the intestinal digestion and absorption process is much more important to assess the potential functionality of a given compound than the simple measurement of its gross concentration. As indicated in the introduction section, a number of different studies developed using *in vitro* models of human digestion have demonstrated that phenolic compounds may strongly interact within the digestive tract both with enzymes and the components of the food matrix, this resulting in important modifications of their potential bioavailability. Regarding the first point, results obtained in the present study evidenced a slight inactivation of seabream proteases in the presence of the phenolic compounds present in WB. Partial inactivation of digestive enzymes in the presence of phenolic compounds has been reported for different enzymes present either in human stomach or intestine (35). Several studies evidence that polyphenols may form complexes by multiple weak interactions (primarily hydrophobic) between amino acid side chains and their aromatic rings. These covalent binding of flavonoids and proteins is usually the result of the reaction between functional groups, such as amino groups of proteins and the quinones formed by oxidation of flavonoids, may prevent the enzymes from interacting with their substrates (36). Some of these interactions have been identified for specific types of catechins and the active catalytic site of trypsin (37). In spite of this, the reduction in the activity of both stomach and intestinal proteases of seabream evidenced in the present study was not high when compared to that produced by specific inhibitors in plant ingredients (26). Hence, it is presumed that this negative effect on digestion associated to the intake of WB (at the levels assayed in the present study) could be easily overcome in the live fish, especially considering that the physiological impact of such partial inactivation of proteases should be modulated by other factors like the total intake of the ingredient or the extension of the feeding period. In addition, other species-specific responses like a compensation of protease inhibition by overproduction of enzymes must be also considered, although it has been reported in salmonids (38) but not in tilapia (39).

The *in vitro* approach used in the present study also evidenced that the potential bioavailability of grape polyphenols contained either in WB or WL was significantly affected by interactions with the feed matrix, as well as by species-specific features of the digestion process (presence/absence of stomach digestion, enzyme profile). Important differences in the profile of the released polyphenols were evidenced as a result of the interaction with the components of the feed matrix. In example, some phenolic compounds as kaempferol, quercetin, catechin and epicatechin showed a significantly reduced release in the presence of food matrix, while others like quercetin 3-glucoside were not affected in the same manner. The feed matrix can be defined as the continuous medium of either cellular origin (i.e., in plant or animal meals) or formed by complex microstructures resulting from processing (i.e., in compound feeds) in which nutrients and bioactive compounds are contained and interact (40). In this sense, it is important to evaluate the potential effects of the food matrix on the bioaccessibility of phenolic compounds and other antioxidants, since only the compounds released and/or absorbed in the intestine are potentially bioavailable and capable of exerting their beneficial effects. The relevance of such interactions has been highlighted by several authors (40–42).

The estimated reduction in the number of phenolic compounds released under simulated digestion was very high in the case of WB (73 and 86% for phenolics for or either mullet and seabream) and also evident in the case of WL (40 and 53%, respectively). As previously indicated, polyphenols have a significant affinity for proteins, this leading to the formation of insoluble complexes of a higher molecular size that can precipitate (43). Nevertheless, they can also interact with other macromolecules present in the feed matrix, like carbohydrates and lipids, reducing absorption of such nutrients and also of the phenolic compounds themselves (42). In fact, it has been demonstrated that the presence of carbohydrates in the digesta determines interactions with some types of phenolic compounds through the formation of protein-tannin-carbohydrate ternary structures (44, 45).There are few studies dealing with the effects of food matrix components and their interactions with specific phenolic compounds according to their typology in human nutrition. It has been reported that isoflavones are the best absorbed dietary flavonoids, flavanols, flavanones and flavonol glycosides are intermediate, whereas proanthocyanins, flavanol gallates and anthocyanins are the worst absorbed (30). However, it is clear that the absorption of specific types of dietary flavonoids may be influenced by the particular types of interactions with the components of the matrix in which they are consumed. As an example, Latruffe et al. (46) reported that quercetin and rutin were more strongly bound to bovine serum albumin than catechin and epicatechin. On the other hand, reductions in the amounts of lysine, cysteine and tryptophan present in soy proteins after interacting with different phenolic compounds like flavonoids, apigenin, kaempferol, quercetin and myricetin have been reported (47).

The differences observed in the present study related to species could be explained considering some key factors characterizing the digestion process in both fish, mainly the absence of an acid stage of hydrolysis in the digestion of mullet and the differences in their digestive biochemistry. In relation to the first point, is worth mentioning that stomach of mullets is formed by a thin-walled cardiac and thick-walled pyloric portion, adapted to function as a mill for feed particles similar to the gizzards of birds, but it does not produce hydrochloric acid or pepsin (48). In contrast, the fully functional stomach present in seabream is able to perform an acid stage of the digestion (49). It has been demonstrated that the chemical structure of the phenolic compounds is closely related to their susceptibility to pH. An acid environment can positively affect the solubilization of some types of phenolic compounds like caffeic, chlorogenic, and gallic acids that are not stable to high pH, while catechin, epigallocatechin, ferulic acid, rutin, and trans-cinnamic acid resist high pH-induced degradation (50). In addition, it is clear that the existence of quantitative and qualitative differences in the type of digestive enzymes between both species may determine a different hydrolysis of the different ingredients of the feed matrix, as well as of the WB and WL, this resulting in a different profile of the released compounds. Finally, is worth to mention that the observed great variations in the patterns of release of different types of phenolic compounds with time suggest an important effect of gut transit rates on the net bioavailability of a given phenolic compound in the live fish. While a fast release could result in a high availability for intestinal absorption, a longer time may determine a lower availability, higher fecal excretion and hence limited biological effects.

## 5. Conclusion

This is, to our knowledge, the first study in which an *in vitro* approach was applied in a similar manner as for humans and terrestrial animals, to assess to what extent the possible complexation of wine polyphenols present in wine by-products with either digestive enzymes or components of the feed matrix could limit their bioaccessibility if included in diets of two different fish species. It is clear that this kind of *in vitro* assays represent a highly valuable tool that can be considered a preliminary step to ascertain results obtained when testing *in vivo* practical use of different types of agri-food by-products that may be used as sources of antioxidant compounds in fish nutrition. Their main advantages arise when considering the wide diversity of phenolic compounds present in any of such products (as occurs in WB and W), as well as the important variations existing between the conditions existing in the digestive tract of fish species with different feeding habits (in terms of digestive enzymes, pH variations or gut transit rates). However, *in vivo* experiments are required in order to validate the results obtained with the present *in vitro* tests since it cannot be ruled out that other factors not identified/tested in the study could affect the rate of release respect to digestion time.

## Data availability statement

The raw data supporting the conclusions of this article will be made available by the authors, without undue reservation.

## Ethics statement

The animal study was reviewed and approved by experimental facilities at the Central Research Services in Marine Cultures (SCI-CM, Operational Code REGA ES11028000312). In addition, all the above-described experiments agree with the Directives of the Spanish Government (RD53/2013) and the European Union Council (2010/63/EU) for the use of animals in research.

## Author contributions

FM-A, JM-S, and JM contributed to the conception and design of the study. FM-A and RL-R organized the database. FM and FM-A performed the statistical analysis. FM-A wrote the first draft of the manuscript. FM, RL-R, JM-S, and JM wrote sections of the manuscript. All authors contributed to manuscript revision, read, and approved the submitted version.

## Funding

RL-R acknowledges to the Andalusian Ministry of Economic Transformation, Industry, Knowledge, and Universities for financial support from “Ayudas para Captación, Incorporación y Movilidad de Capital Humano de I + D + I (PAIDI 2020).” This research was developed within the Project UBAGALAC, funded by the Junta de Andalucía Proyectos Reto 2020 (P20-00923).

## Conflict of interest

The authors declare that the research was conducted in the absence of any commercial or financial relationships that could be construed as a potential conflict of interest.

## Publisher’s note

All claims expressed in this article are solely those of the authors and do not necessarily represent those of their affiliated organizations, or those of the publisher, the editors and the reviewers. Any product that may be evaluated in this article, or claim that may be made by its manufacturer, is not guaranteed or endorsed by the publisher.

## References

[1] Arvanitoyannis IS Ladas D Mavromatis A. Potential uses and applications of treated wine waste: a review. Int J Food Sci Technol. 41:475–87. doi: 10.1111/j.1365-2621.2005.01111.x

[2] Galanakis CM. Recovery of high added-value components from food wastes: conventional, emerging technologies and commercialized applications. Trends Food Sci Technol. (2012) 26:68–87. doi: 10.1016/j.tifs.2012.03.003

[3] Galanakis CM Schieber A. Recovery and utilization of valuable compounds from food processing by-products. Food Res Int. 65:299–300. doi: 10.1016/j.foodres.2014.11.019

[4] Hang YD. Recovery of food ingredients from grape pomace. Process Biochem. (1988) 23:2–4.

[5] Prokopov T Goranova Z Baeva M Slavov A Galanakis CM. Effects of powder from white cabbage outer leaves on sponge cake quality. Int Agrophys. 29:493–13. doi: 10.1515/intag-2015-0055

[6] Costa M Alfaia CM Lopes PA Pestana JM Prates JA. Grape by-products as feedstuff for pig and poultry production. Animals. (2022) 12:2239. doi: 10.3390/ani12172239, PMID: 36077957 PMC9454619

[7] Chedea VS Palade LM Pelmus RS Dragomir C Taranu I. Red grape pomace rich in polyphenols diet increases the antioxidant status in key organs—kidneys, liver, and spleen of piglets. Animals. (2019) 9:149. doi: 10.3390/ani9040149, PMID: 30959837 PMC6524171

[8] Turcu R Margareta O Criste RD Mariana R Tatiana P Soica C. The effect of using grape seeds meal as natural antioxidant in broiler diets enriched in fatty acids, on meat quality. J Hygien Eng Design. (2019) 29:14–20.

[9] Souza CF Baldissera MD Descovi SN Zeppenfeld CC Verdi CM Santos RCV. Grape pomace flour alleviates *Pseudomonas aeruginosa*-induced hepatic oxidative stress in grass carp by improving antioxidant defense. Microb Pathog. (2019) 129:271–6. doi: 10.1016/j.micpath.2019.02.024 30802491

[10] Baldissera MD Souza CF Descovi SN Verdi CM Zeppenfeld CC da Silva AS. Grape pomace flour ameliorates *Pseudomonas aeruginosa*-induced bioenergetic dysfunction in gills of grass carp. Aquaculture. (2019) 506:359–6. doi: 10.1016/j.aquaculture.2019.03.065

[11] Peña E Badillo-Zapata D Viana MT Correa-Reyes G. Use of grape pomace in formulated feed for the rainbow trout fry, *Oncorhynchus mykiss* (Walbaum, 1792). J World Aquacult Soc. (2020) 51:542–13. doi: 10.1111/jwas.12669

[12] Pulgar R Mandakovic D Salgado P Venegas L Ortiz D Peña-Neira Á. Micro-encapsulated grape pomace extract (MGPE) as a feed additive improves growth performance, antioxidant capacity, and shifts the gut microbiome of rainbow trout. Aquaculture. (2021) 544:737129. doi: 10.1016/j.aquaculture.2021.737129

[13] Mandalari G Vardakou M Faulks R Bisignano C Martorana M Smeriglio A. Food matrix effects of polyphenol bioaccessibility from almond skin during simulated human digestion. Nutrients. (2016) 8. doi: 10.3390/nu8090568, PMID: 27649239 PMC5037553

[14] Seczyk Ł Gawlik-Dziki U Świeca M. Influence of phenolic-food matrix interactions on *in vitro* bioaccessibility of selected phenolic compounds and nutrients digestibility in fortified white bean paste. Antioxidants. (2021) 10:1825. doi: 10.3390/antiox10111825, PMID: 34829697 PMC8614679

[15] Sengul H Surek E Nilufer-Erdil D. Investigating the effects of food matrix and food components on bioaccessibility of pomegranate (*Punica granatum*) phenolics and anthocyanins using an in-vitro gastrointestinal digestion model. Food Res Int. (2014) 62:1069–79. doi: 10.1016/j.foodres.2014.05.055

[16] Tagliazucchi D Verzelloni E Bertolini D Conte A. *In vitro* bio-accessibility and antioxidant activity of grape polyphenols. Food Chem. (2010) 120:599–6. doi: 10.1016/j.foodchem.2009.10.030

[17] Alarcón FJ Moyano FJ Díaz M. Evaluation of different protein sources of aquafeeds by an optimised pH-stat system. J Sci Food Agric. (2002) 82:697–4. doi: 10.1002/jsfa.1100

[18] Hamdan M Moyano FJ Schuhardt D. Optimization of a gastrointestinal model applicable to the evaluation of protein bioaccessibility in fish feeds. J Sci Food Agric. (2009) 89:1195–01. doi: 10.1002/jsfa.3574

[19] Gilannejad N Martínez-Rodríguez G Yúfera M Moyano FJ. Estimating the effect of different factors on the digestive bioaccessibility of protein by the Senegalese sole (*Solea senegalensis*); combination of response surface methodology and *in vitro* assays. Aquaculture. (2017) 477:28–34. doi: 10.1016/j.aquaculture.2017.04.037

[20] Nogueira WV Moyano FJ García MJA Tesser MB Buffon JG. Preliminary assessment of bioaccessibility of aflatoxin B1 in fish. Aquac Int. (2022) 30:1315–25. doi: 10.1007/s10499-022-00860-4

[21] Revilla E Ryan JM. Analysis of several phenolic compounds with potential antioxidant properties in grape extracts and wines by high-performance liquid chromatography–photodiode array detection without sample preparation. J Chromatogr. (2000) 881:461–9. doi: 10.1016/S0021-9673(00)00269-7 10905728

[22] Sun J Liang F Bin Y Ping L Duan C. Screening non-colored Phenolics in red wines using liquid chromatography/ultraviolet and mass spectrometry/mass spectrometry libraries. Molecules. (2007) 12:679–3. doi: 10.3390/12030679, PMID: 17851421 PMC6149347

[23] Liu S Marsol-Vall A Laaksonen O Kortesniemi M Yang B. Characterization and quantification of nonanthocyanin phenolic compounds in white and blue bilberry (*Vaccinium myrtillus*) juices and wines using UHPLC-DAD−ESI-QTOF-MS and UHPLC-DAD. J Agric Food Chem. (2020) 68:7734–44. doi: 10.1021/acs.jafc.0c02842, PMID: 32609509 PMC7497633

[24] López-Gutiérrez N Romero-González R Martínez L Garrido A. Determination of polyphenols in grape-based nutraceutical products using high resolution mass spectrometry. Food Sci Technol. (2016) 71:249–9. doi: 10.1016/j.lwt.2016.03.037

[25] Vrhovsek U Masuero D Gasperotti M Franceschi P Caputi L Viola R. A versatile targeted metabolomics method for the rapid quantification of multiple classes of Phenolics in fruits and beverages. J Agric Food Chem. (2012) 60:8831–40. doi: 10.1021/jf2051569, PMID: 22468648

[26] Moyano FJ Martínez I Díaz M Alarcón FJ. Inhibition of digestive proteases by vegetable meals in three fish species; seabream (*Sparus aurata*), tilapia (*Oreochromis niloticus*) and African sole (*Solea senegalensis*). Comp Biochem Physiol. (1998) 122B:327–2.

[27] Anson ML. The estimation of pepsin, trypsin, papain, and cathepsin with hemoglobin. J Gen Physiol. (1938) 22:79–89. doi: 10.1085/jgp.22.1.79, PMID: 19873094 PMC2213732

[28] Walter HE “Proteinases: methods with haemoglobin, casein and azocoll as substrates,” in Methods of enzymatic analysis. ed. Bergmeyer H. U. (Weinheim: Verlag Chemie). (1984) V:270–277.

[29] Morales GA Moyano FJ. Application of an *in vitro* gastrointestinal model to evaluate nitrogen and phosphorus bioaccessibility and bioavailability in fish feed ingredients. Aquaculture. (2010) 306:244–1. doi: 10.1016/j.aquaculture.2010.05.014

[30] Beres C Costa GNS Cabezudo I da Silva-James NK Teles ASC Cruz APG. Towards integral utilization of grape pomace from winemaking process: a review. Waste Manag. (2017) 68:581–4. doi: 10.1016/j.wasman.2017.07.017, PMID: 28734610

[31] González-Manzano S Rivas-Gonzalo JC Santos-Buelga C. Extraction of flavan-3-ols from grape seed and skin into wine using simulated maceration. Anal Chim Acta. (2004) 513:283–9. doi: 10.1016/j.aca.2003.10.019

[32] Makris DP Kallithraka S Kefalas P. Flavonols in grapes, grape products and wines: burden, profile and influential parameters. J Food Compos Anal. (2006) 19:396–4. doi: 10.1016/j.jfca.2005.10.003

[33] Mena P Ascacio-Valdés JA Gironés-Vilaplana A del Rio D Moreno DA García-Viguera C. Assessment of pomegranate wine lees as a valuable source for the recovery of (poly)phenolic compounds. Food Chem. (2014) 145:327–4. doi: 10.1016/j.foodchem.2013.08.039 24128485

[34] Jara-Palacios MJ. Wine lees as a source of antioxidant compounds. Antioxidants. (2019) 8:1–9. doi: 10.3390/antiox8020045, PMID: 30781536 PMC6406673

[35] Martínez-Gonzalez A Díaz-Sánchez A de la Rosa L Vargas-Requena C Bustos-Jaimes I Alvarez-Parrilla E. Polyphenolic compounds and digestive enzymes: *in vitro* non-covalent interactions. Molecules. (2017) 22:669. doi: 10.3390/molecules22040669 28441731 PMC6154557

[36] Barrett A Farhadi N Smith T. Slowing starch digestion and inhibiting digestive enzyme activity using plant flavanols/tannins. A review of efficacy and mechanisms. Food Sci Technol. (2018) 87:394–9. doi: 10.1016/j.lwt.2017.09.002

[37] Cui F Yang K Li Y. Investigate the binding of catechins to trypsin using docking and molecular dynamics simulation. PLoS One. (2015) 10. doi: 10.1371/journal.pone.0125848, PMID: 25938485 PMC4418572

[38] Haard NF Dimes LE Arndt RE Dong FM. Estimation of protein digestibility IV. Digestive proteinases from the pyloric caeca of Coho Salmon (*Oncorhynchus kisutch*) fed diets containing soybean meal. Comp Biochem Physiol B: Biochem Mol Biol. (1996) 115:533–40. doi: 10.1016/S0305-0491(96)00189-7

[39] Anderson J Capper BS Bromage NR. Measurement and prediction of digestible energy values in feedstuffs for the herbivorous fish tilapia (*Oreochromis niloticus Linn*.). Br J Nutr. (1991) 66:37–48. doi: 10.1079/bjn19910007, PMID: 1931904

[40] Aguilera JM. The food matrix: implications in processing, nutrition and health. Crit Rev Food Sci Nutr. (2019) 59:3612–29. doi: 10.1080/10408398.2018.1502743, PMID: 30040431

[41] Oliveira A Amaro AL Pintado M. Impact of food matrix components on nutritional and functional properties of fruit-based products. Curr Opin Food Sci. (2018) 22:153–9. doi: 10.1016/j.cofs.2018.04.002

[42] Zhang H Yu D Sun J Liu X Jiang L Guo H. Interaction of plant phenols with food macronutrients: characterisation and nutritional-physiological consequences. Nutrition research reviews (2014), 27, 1–15. Cambridge: Cambridge University Press. 24169001 10.1017/S095442241300019X

[43] Ozdal T Capanoglu E Altay F. A review on protein-phenolic interactions and associated changes. Food Res Int. (2013) 51:954–13. doi: 10.1016/j.foodres.2013.02.009

[44] De Freitas V Carvalho E Mateus N. Study of carbohydrate influence on protein-tannin aggregation by nephelometry. Food Chem. (2003) 81:503–9. doi: 10.1016/S0308-8146(02)00479-X

[45] Mateus N Carvalho E Luís C de Freitas V. Influence of the tannin structure on the disruption effect of carbohydrates on protein-tannin aggregates. Anal Chim Acta. (2004) 513:135–13. doi: 10.1016/j.aca.2003.08.072

[46] Latruffe N Menzel M Delmas D Buchet R Lançon A. Compared binding properties between resveratrol and other polyphenols to plasmatic albumin: consequences for the health protecting effect of dietary plant microcomponents. Molecules. (2014) 19:17066–77. doi: 10.3390/molecules191117066 25347454 PMC6270862

[47] Rawel HM Rte Czajka D Rohn S Rgen Kroll J. Interactions of different phenolic acids and flavonoids with soy proteins. Int J Biol Macromol. (2002) 30:137–13. doi: 10.1016/S0141-8130(02)00016-8, PMID: 12063116

[48] Payne AI. Gut pH and digestive strategies in estuarine grey mullet (*Mugilidae*) and tilapia (*Cichlidae*). J Fish Biol. (1978) 13:627–9. doi: 10.1111/j.1095-8649.1978.tb03476.x

[49] Márquez L Robles R Morales G Moyano FJ. Gut pH as a limiting factor for digestive proteolysis in cultured juveniles of the gilthead sea bream (*Sparus aurata*). Fish Physiol Biochem. (2012) 38:859–9. doi: 10.1007/s10695-011-9573-1, PMID: 22086356

[50] Friedman M Jurgens HS. Effect of pH on the stability of plant phenolic compounds. J Agric Food Chem. (2000) 48:2101–10. doi: 10.1021/jf990489j 10888506

[51] Kunitz M. Crystalline soybean trypsin inhibitor: II. General properties. J Gen Physiol. (1947) 30:291–13. doi: 10.1085/jgp.30.4.291, PMID: 19873496 PMC2142836

[52] Re R Pellegrini N Proteggente A Pannala A Yang M Rice-Evans C. Antioxidant activity and improved ABTS radical cation decolorization assay, in Free radical biology and medicine. Elsevier. (1999) 26:1231–1237. 10381194 10.1016/s0891-5849(98)00315-3

